# 2,3-Diamino­pyridinium 3-chloro­benzo­ate–3-chloro­benzoic acid (1/1)

**DOI:** 10.1107/S1600536811050422

**Published:** 2011-11-30

**Authors:** Madhukar Hemamalini, Jia Hao Goh, Hoong-Kun Fun

**Affiliations:** a X-ray Crystallography Unit, School of Physics, Universiti Sains Malaysia, 11800 USM, Penang, Malaysia

## Abstract

The asymmetric unit of the title compound, C_5_H_8_N_3_^+^·C_7_H_4_ClO_2_^−^·C_7_H_5_ClO_2_, contains an ion pair and a 3-chloro­benzoic acid mol­ecule. In the cation, the pyridine N atom is protonated. In the crystal, the components are connected *via* N—H⋯O, O—H⋯O and C—H⋯O hydrogen bonds, thereby forming sheets lying parallel to (100).

## Related literature

For further details on 2-amino­pyridine, see: Bis & Zaworotko (2005 ▶); Bis *et al.* (2006 ▶). For general background to inter­molecular inter­actions, see: Desiraju (2001 ▶); Haddad & Willett (2001 ▶); Willett *et al.* (2003 ▶). For bond-length data, see: Allen *et al.* (1987 ▶). For the stability of the temperature controller used in the data collection, see: Cosier & Glazer (1986 ▶).
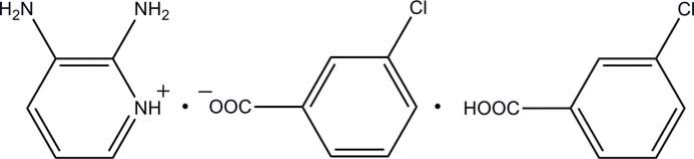


## Experimental

### 

#### Crystal data

C_5_H_8_N_3_^+^·C_7_H_4_ClO_2_^−^·C_7_H_5_ClO_2_
*M* *_r_* = 422.26
Orthorhombic, 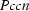

*a* = 33.3187 (7) Å
*b* = 8.6628 (2) Å
*c* = 13.1811 (2) Å
*V* = 3804.50 (13) Å^3^
*Z* = 8
Mo *K*α radiation
μ = 0.37 mm^−1^
*T* = 100 K
0.44 × 0.19 × 0.05 mm

#### Data collection

Bruker SMART APEXII CCD diffractometer
Absorption correction: multi-scan (*SADABS*; Bruker, 2009 ▶) *T*_min_ = 0.854, *T*_max_ = 0.980
25995 measured reflections
5596 independent reflections
3717 reflections with *I* > 2σ(*I*)
*R*_int_ = 0.079

#### Refinement

*R*[*F* ^2^ > 2σ(*F* ^2^)] = 0.062
*wR*(*F* ^2^) = 0.149
*S* = 1.02
5596 reflections
254 parameters
H-atom parameters constrained
Δρ_max_ = 0.65 e Å^−3^
Δρ_min_ = −0.57 e Å^−3^

### 

Data collection: *APEX2* (Bruker, 2009 ▶); cell refinement: *SAINT* (Bruker, 2009 ▶); data reduction: *SAINT*; program(s) used to solve structure: *SHELXTL* (Sheldrick, 2008 ▶); program(s) used to refine structure: *SHELXTL*; molecular graphics: *SHELXTL*; software used to prepare material for publication: *SHELXTL* and *PLATON* (Spek, 2009 ▶).

## Supplementary Material

Crystal structure: contains datablock(s) global, I. DOI: 10.1107/S1600536811050422/hb6530sup1.cif

Structure factors: contains datablock(s) I. DOI: 10.1107/S1600536811050422/hb6530Isup2.hkl

Supplementary material file. DOI: 10.1107/S1600536811050422/hb6530Isup3.cml

Additional supplementary materials:  crystallographic information; 3D view; checkCIF report

## Figures and Tables

**Table 1:** Hydrogen-bond geometry (Å, °)

*D*—H⋯*A*	*D*—H	H⋯*A*	*D*⋯*A*	*D*—H⋯*A*
N1—H1⋯O3^i^	0.86	2.01	2.824 (3)	157
N1—H1⋯O4^i^	0.86	2.44	3.171 (3)	144
O1—H1*A*⋯O4	0.82	1.77	2.582 (2)	169
N2—H2*A*⋯O4^i^	0.86	2.07	2.886 (3)	158
N2—H2*B*⋯O3^ii^	0.86	2.18	3.021 (3)	168
N3—H3*A*⋯O2	0.86	2.25	3.011 (3)	147
N3—H3*B*⋯O3^ii^	0.86	2.22	3.046 (3)	161
C3—H3⋯O2	0.93	2.36	3.141 (3)	142

Symmetry codes: (i) 
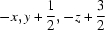
; (ii) 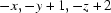.

**Fig. 1.**
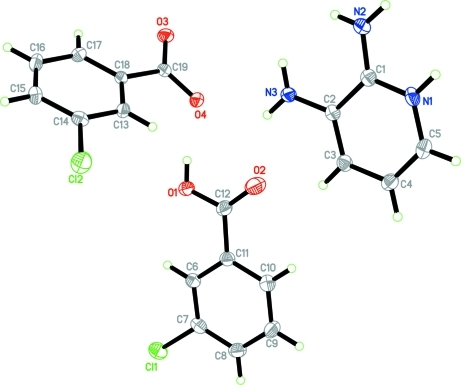
The asymmetric unit of the title compound, showing 50% probability displacement ellipsoids.

**Fig. 2.**
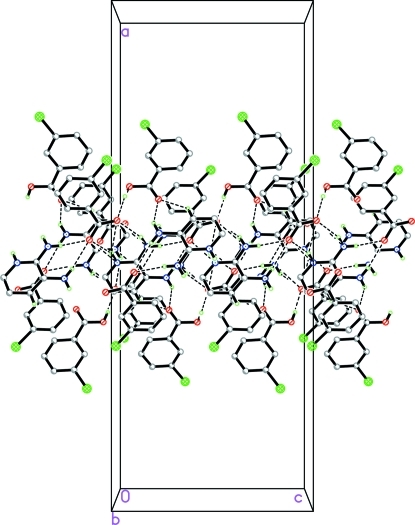
The crystal packing of title compound (I).

## supplementary crystallographic information

### Comment

2-Aminopyridine is one of the most frequently used synthons in supramolecular chemistry based on hydrogen bonds (Bis & Zaworotko, 2005; Bis *et al.*, 2006). In the crystals of such compounds, weak intermolecular interactions involving halide ions, halogen–halide interactions, as well as π···π stacking effects, are found to play an important role in the organization of the structural units (Desiraju, 2001; Haddad & Willett, 2001; Willett *et al.*, 2003). In order to study some interesting hydrogen bonding interactions, the synthesis and structure of the title compound, (I), is presented here.

The asymmetric unit of (I) (Fig 1), contains a protonated 2,3-diamino pyridinium cation, a 3-chlorobenzoate anion and a neutral 3-chlorobenzoic acid. In the 2,3-diaminopyridinium cation, the protonated N1 atom has lead to a slight increase in the C1—N1—C5 angle to 124.0 (2)°. The dihedral angle between the pyridine (N1/C1–C5) and each of the two phenyl (C6–C11/C13–C18) rings are 8.68 (12) and 75.42 (12)°, respectively. The bond lengths (Allen *et al.*, 1987) and angles are normal.

In the crystal of (I), (Fig. 2), the ion-pairs and the neutral acid molecules are connected *via* N—H···O, O—H···N and C—H···O hydrogen bonds forming two-dimensional networks parallel to (1 0 0)-plane.

### Experimental

Hot methanol solutions (20 ml) of 2,3-diaminopyridine (27 mg, Aldrich) and 3-chlorobenzoic acid (39 mg, Merck) were mixed and warmed over a heated magnetic stirrer for 5 minutes. The resulting solution was allowed to cool slowly at room temperature.  Brown plates of (I) appeared from the mother liquor after a few days.

### Refinement

All hydrogen atoms were positioned geometrically [N–H = 0.86 and C–H = 0.93 Å] and were refined using a riding model, with *U*_iso_(H) = 1.2 or 1.5 *U*_eq_(C).  A rotating group model was applied to the methyl groups.

### Crystal data

**Table:**

C_5_H_8_N_3_^+^·C_7_H_4_ClO_2_^−^·C_7_H_5_ClO_2_	*F*(000) = 1744
*M**_r_* = 422.26	*D*_x_ = 1.474 Mg m^−^^3^
Orthorhombic, *P**c**c**n*	Mo *K*α radiation, λ = 0.71073 Å
Hall symbol: -P 2ab 2ac	Cell parameters from 3141 reflections
*a* = 33.3187 (7) Å	θ = 2.9–25.1°
*b* = 8.6628 (2) Å	µ = 0.37 mm^−^^1^
*c* = 13.1811 (2) Å	*T* = 100 K
*V* = 3804.50 (13) Å^3^	Plate, brown
*Z* = 8	0.44 × 0.19 × 0.05 mm

### Data collection

**Table:**

Bruker SMART APEXII CCD diffractometer	5596 independent reflections
Radiation source: fine-focus sealed tube	3717 reflections with *I* > 2σ(*I*)
graphite	*R*_int_ = 0.079
φ and ω scans	θ_max_ = 30.1°, θ_min_ = 2.4°
Absorption correction: multi-scan (*SADABS*; Bruker, 2009)	*h* = −46→46
*T*_min_ = 0.854, *T*_max_ = 0.980	*k* = −8→12
25995 measured reflections	*l* = −14→18

### Refinement

**Table:**

Refinement on *F*^2^	Primary atom site location: structure-invariant direct methods
Least-squares matrix: full	Secondary atom site location: difference Fourier map
*R*[*F*^2^ > 2σ(*F*^2^)] = 0.062	Hydrogen site location: inferred from neighbouring sites
*wR*(*F*^2^) = 0.149	H-atom parameters constrained
*S* = 1.02	*w* = 1/[σ^2^(*F*_o_^2^) + (0.0532*P*)^2^ + 4.6426*P*] where *P* = (*F*_o_^2^ + 2*F*_c_^2^)/3
5596 reflections	(Δ/σ)_max_ < 0.001
254 parameters	Δρ_max_ = 0.65 e Å^−^^3^
0 restraints	Δρ_min_ = −0.57 e Å^−^^3^

### Special details

**Table:**

Experimental. The crystal was placed in the cold stream of an Oxford Cryosystems Cobra open-flow nitrogen cryostat (Cosier & Glazer, 1986) operating at 100.0 (1) K.
Geometry. All s.u.'s (except the s.u. in the dihedral angle between two l.s. planes) are estimated using the full covariance matrix. The cell s.u.'s are taken into account individually in the estimation of s.u.'s in distances, angles and torsion angles; correlations between s.u.'s in cell parameters are only used when they are defined by crystal symmetry. An approximate (isotropic) treatment of cell s.u.'s is used for estimating s.u.'s involving l.s. planes.
Refinement. Refinement of F^2^ against ALL reflections. The weighted R-factor wR and goodness of fit S are based on F^2^, conventional R-factors R are based on F, with F set to zero for negative F^2^. The threshold expression of F^2^ > 2σ(F^2^) is used only for calculating R-factors(gt) etc. and is not relevant to the choice of reflections for refinement. R-factors based on F^2^ are statistically about twice as large as those based on F, and R- factors based on ALL data will be even larger.

### Fractional atomic coordinates and isotropic or equivalent isotropic displacement parameters (Å^2^)

**Table:**

	*x*	*y*	*z*	*U*_iso_*/*U*_eq_	
N1	0.01367 (6)	0.6953 (2)	0.51452 (16)	0.0206 (5)	
H1	−0.0050	0.7511	0.4885	0.025*	
N2	−0.01884 (6)	0.7200 (3)	0.66837 (17)	0.0234 (5)	
H2A	−0.0373	0.7729	0.6389	0.028*	
H2B	−0.0203	0.7020	0.7324	0.028*	
N3	0.04450 (6)	0.5583 (2)	0.76248 (15)	0.0194 (4)	
H3A	0.0641	0.5096	0.7905	0.023*	
H3B	0.0255	0.5959	0.7993	0.023*	
C1	0.01206 (7)	0.6658 (3)	0.61463 (19)	0.0184 (5)	
C2	0.04354 (7)	0.5763 (3)	0.65881 (18)	0.0159 (5)	
C3	0.07273 (7)	0.5196 (3)	0.59485 (18)	0.0191 (5)	
H3	0.0931	0.4581	0.6214	0.023*	
C4	0.07254 (8)	0.5523 (3)	0.49033 (19)	0.0207 (5)	
H4	0.0924	0.5126	0.4482	0.025*	
C5	0.04304 (8)	0.6425 (3)	0.4521 (2)	0.0222 (5)	
H5	0.0429	0.6682	0.3836	0.027*	
Cl1	0.27106 (2)	−0.04829 (9)	0.85137 (6)	0.03177 (18)	
O1	0.13896 (5)	0.2618 (2)	0.91982 (13)	0.0243 (4)	
H1A	0.1189	0.3054	0.9414	0.036*	
O2	0.11287 (7)	0.3315 (3)	0.77151 (16)	0.0452 (6)	
C6	0.20367 (7)	0.1198 (3)	0.8287 (2)	0.0207 (5)	
H6	0.2023	0.1203	0.8991	0.025*	
C7	0.23479 (7)	0.0453 (3)	0.7791 (2)	0.0229 (5)	
C8	0.23713 (8)	0.0418 (3)	0.6741 (2)	0.0265 (6)	
H8	0.2580	−0.0104	0.6421	0.032*	
C9	0.20807 (8)	0.1168 (3)	0.6175 (2)	0.0281 (6)	
H9	0.2095	0.1156	0.5470	0.034*	
C10	0.17703 (8)	0.1931 (3)	0.6651 (2)	0.0246 (6)	
H10	0.1577	0.2441	0.6268	0.030*	
C11	0.17461 (7)	0.1938 (3)	0.77091 (19)	0.0194 (5)	
C12	0.13958 (8)	0.2704 (3)	0.8195 (2)	0.0229 (6)	
Cl2	0.18144 (2)	0.80228 (9)	1.00830 (5)	0.03097 (18)	
O3	0.03184 (5)	0.3859 (2)	1.11579 (14)	0.0232 (4)	
O4	0.07032 (5)	0.3671 (2)	0.97878 (14)	0.0231 (4)	
C13	0.12004 (7)	0.6069 (3)	1.04635 (19)	0.0177 (5)	
H13	0.1203	0.5860	0.9771	0.021*	
C14	0.14733 (7)	0.7093 (3)	1.0881 (2)	0.0206 (5)	
C15	0.14826 (8)	0.7410 (3)	1.1909 (2)	0.0234 (6)	
H15	0.1671	0.8093	1.2175	0.028*	
C16	0.12051 (8)	0.6688 (3)	1.2534 (2)	0.0249 (6)	
H16	0.1208	0.6884	1.3228	0.030*	
C17	0.09240 (8)	0.5677 (3)	1.21324 (19)	0.0208 (5)	
H17	0.0736	0.5214	1.2557	0.025*	
C18	0.09208 (7)	0.5352 (3)	1.11004 (18)	0.0157 (5)	
C19	0.06258 (7)	0.4214 (3)	1.06588 (19)	0.0182 (5)	

### Atomic displacement parameters (Å^2^)

**Table:**

	*U*^11^	*U*^22^	*U*^33^	*U*^12^	*U*^13^	*U*^23^
N1	0.0220 (10)	0.0193 (11)	0.0205 (11)	0.0026 (9)	−0.0071 (8)	0.0014 (9)
N2	0.0206 (11)	0.0263 (13)	0.0232 (12)	0.0072 (9)	−0.0027 (9)	−0.0019 (9)
N3	0.0178 (10)	0.0243 (11)	0.0161 (10)	0.0047 (9)	0.0005 (8)	0.0021 (8)
C1	0.0200 (12)	0.0145 (12)	0.0209 (13)	−0.0017 (9)	−0.0026 (9)	−0.0029 (9)
C2	0.0163 (11)	0.0129 (11)	0.0185 (12)	−0.0031 (9)	−0.0010 (9)	0.0008 (9)
C3	0.0196 (12)	0.0198 (13)	0.0178 (12)	0.0008 (10)	−0.0011 (9)	0.0032 (9)
C4	0.0214 (12)	0.0213 (13)	0.0194 (12)	−0.0025 (10)	0.0021 (9)	−0.0025 (10)
C5	0.0272 (13)	0.0232 (14)	0.0162 (12)	−0.0037 (11)	−0.0014 (10)	−0.0015 (10)
Cl1	0.0250 (3)	0.0342 (4)	0.0360 (4)	0.0090 (3)	−0.0026 (3)	0.0002 (3)
O1	0.0217 (9)	0.0332 (11)	0.0181 (9)	0.0076 (8)	0.0005 (7)	−0.0003 (8)
O2	0.0437 (13)	0.0710 (17)	0.0209 (11)	0.0359 (12)	0.0007 (9)	0.0056 (11)
C6	0.0183 (11)	0.0224 (13)	0.0213 (13)	−0.0016 (10)	0.0005 (9)	−0.0004 (10)
C7	0.0176 (12)	0.0217 (13)	0.0293 (14)	0.0005 (10)	−0.0023 (10)	−0.0007 (11)
C8	0.0216 (13)	0.0288 (15)	0.0292 (15)	0.0001 (11)	0.0046 (10)	−0.0069 (12)
C9	0.0288 (14)	0.0369 (17)	0.0185 (13)	−0.0045 (13)	0.0034 (11)	−0.0033 (12)
C10	0.0229 (13)	0.0285 (14)	0.0224 (13)	−0.0005 (11)	−0.0017 (10)	−0.0006 (11)
C11	0.0181 (12)	0.0193 (12)	0.0209 (12)	0.0003 (10)	0.0008 (9)	−0.0019 (10)
C12	0.0241 (13)	0.0247 (14)	0.0199 (13)	0.0053 (11)	0.0000 (10)	−0.0015 (10)
Cl2	0.0307 (3)	0.0345 (4)	0.0277 (4)	−0.0138 (3)	0.0060 (3)	−0.0006 (3)
O3	0.0162 (8)	0.0277 (10)	0.0256 (10)	−0.0026 (8)	0.0037 (7)	−0.0052 (8)
O4	0.0180 (9)	0.0303 (11)	0.0209 (9)	−0.0013 (8)	−0.0002 (7)	−0.0093 (8)
C13	0.0187 (11)	0.0197 (13)	0.0146 (11)	0.0023 (10)	−0.0021 (9)	−0.0020 (9)
C14	0.0154 (11)	0.0230 (13)	0.0234 (13)	−0.0001 (10)	0.0016 (9)	0.0018 (10)
C15	0.0214 (12)	0.0259 (14)	0.0229 (13)	−0.0039 (11)	−0.0042 (10)	−0.0062 (11)
C16	0.0258 (13)	0.0319 (15)	0.0171 (12)	−0.0029 (12)	−0.0020 (10)	−0.0059 (10)
C17	0.0198 (12)	0.0266 (14)	0.0161 (12)	0.0006 (10)	0.0023 (9)	−0.0024 (10)
C18	0.0146 (11)	0.0165 (12)	0.0158 (11)	0.0031 (9)	−0.0019 (8)	−0.0015 (9)
C19	0.0155 (11)	0.0202 (13)	0.0188 (12)	0.0029 (10)	−0.0019 (9)	−0.0017 (9)

### Geometric parameters (Å, °)

**Table:**

N1—C1	1.345 (3)	C7—C8	1.387 (4)
N1—C5	1.358 (3)	C8—C9	1.384 (4)
N1—H1	0.8600	C8—H8	0.9300
N2—C1	1.335 (3)	C9—C10	1.379 (4)
N2—H2A	0.8600	C9—H9	0.9300
N2—H2B	0.8600	C10—C11	1.397 (4)
N3—C2	1.376 (3)	C10—H10	0.9300
N3—H3A	0.8600	C11—C12	1.488 (3)
N3—H3B	0.8600	Cl2—C14	1.745 (3)
C1—C2	1.428 (3)	O3—C19	1.256 (3)
C2—C3	1.378 (3)	O4—C19	1.267 (3)
C3—C4	1.407 (3)	C13—C14	1.384 (3)
C3—H3	0.9300	C13—C18	1.399 (3)
C4—C5	1.353 (4)	C13—H13	0.9300
C4—H4	0.9300	C14—C15	1.383 (4)
C5—H5	0.9300	C15—C16	1.387 (4)
Cl1—C7	1.739 (3)	C15—H15	0.9300
O1—C12	1.324 (3)	C16—C17	1.388 (4)
O1—H1A	0.8200	C16—H16	0.9300
O2—C12	1.214 (3)	C17—C18	1.389 (3)
C6—C7	1.385 (4)	C17—H17	0.9300
C6—C11	1.388 (3)	C18—C19	1.509 (3)
C6—H6	0.9300		
			
C1—N1—C5	124.0 (2)	C10—C9—C8	120.3 (3)
C1—N1—H1	118.0	C10—C9—H9	119.9
C5—N1—H1	118.0	C8—C9—H9	119.9
C1—N2—H2A	120.0	C9—C10—C11	120.0 (3)
C1—N2—H2B	120.0	C9—C10—H10	120.0
H2A—N2—H2B	120.0	C11—C10—H10	120.0
C2—N3—H3A	120.0	C6—C11—C10	120.3 (2)
C2—N3—H3B	120.0	C6—C11—C12	121.1 (2)
H3A—N3—H3B	120.0	C10—C11—C12	118.5 (2)
N2—C1—N1	119.0 (2)	O2—C12—O1	122.3 (2)
N2—C1—C2	122.7 (2)	O2—C12—C11	123.0 (2)
N1—C1—C2	118.3 (2)	O1—C12—C11	114.6 (2)
N3—C2—C3	123.4 (2)	C14—C13—C18	118.9 (2)
N3—C2—C1	118.9 (2)	C14—C13—H13	120.6
C3—C2—C1	117.5 (2)	C18—C13—H13	120.6
C2—C3—C4	121.6 (2)	C15—C14—C13	122.1 (2)
C2—C3—H3	119.2	C15—C14—Cl2	118.9 (2)
C4—C3—H3	119.2	C13—C14—Cl2	119.0 (2)
C5—C4—C3	119.0 (2)	C14—C15—C16	118.5 (2)
C5—C4—H4	120.5	C14—C15—H15	120.7
C3—C4—H4	120.5	C16—C15—H15	120.7
C4—C5—N1	119.5 (2)	C15—C16—C17	120.5 (2)
C4—C5—H5	120.3	C15—C16—H16	119.7
N1—C5—H5	120.3	C17—C16—H16	119.7
C12—O1—H1A	109.5	C16—C17—C18	120.4 (2)
C7—C6—C11	118.6 (2)	C16—C17—H17	119.8
C7—C6—H6	120.7	C18—C17—H17	119.8
C11—C6—H6	120.7	C17—C18—C13	119.5 (2)
C6—C7—C8	121.5 (2)	C17—C18—C19	121.0 (2)
C6—C7—Cl1	118.7 (2)	C13—C18—C19	119.5 (2)
C8—C7—Cl1	119.8 (2)	O3—C19—O4	123.3 (2)
C9—C8—C7	119.2 (2)	O3—C19—C18	119.3 (2)
C9—C8—H8	120.4	O4—C19—C18	117.4 (2)
C7—C8—H8	120.4		
			
C5—N1—C1—N2	−178.1 (2)	C9—C10—C11—C12	−176.9 (3)
C5—N1—C1—C2	1.4 (4)	C6—C11—C12—O2	−177.4 (3)
N2—C1—C2—N3	−7.0 (4)	C10—C11—C12—O2	0.5 (4)
N1—C1—C2—N3	173.5 (2)	C6—C11—C12—O1	0.2 (4)
N2—C1—C2—C3	176.6 (2)	C10—C11—C12—O1	178.0 (2)
N1—C1—C2—C3	−3.0 (3)	C18—C13—C14—C15	1.1 (4)
N3—C2—C3—C4	−174.2 (2)	C18—C13—C14—Cl2	−178.48 (19)
C1—C2—C3—C4	2.1 (4)	C13—C14—C15—C16	−0.8 (4)
C2—C3—C4—C5	0.4 (4)	Cl2—C14—C15—C16	178.8 (2)
C3—C4—C5—N1	−2.0 (4)	C14—C15—C16—C17	−0.3 (4)
C1—N1—C5—C4	1.1 (4)	C15—C16—C17—C18	1.1 (4)
C11—C6—C7—C8	−0.8 (4)	C16—C17—C18—C13	−0.8 (4)
C11—C6—C7—Cl1	−179.7 (2)	C16—C17—C18—C19	177.9 (2)
C6—C7—C8—C9	1.1 (4)	C14—C13—C18—C17	−0.3 (4)
Cl1—C7—C8—C9	−180.0 (2)	C14—C13—C18—C19	−179.1 (2)
C7—C8—C9—C10	−0.5 (4)	C17—C18—C19—O3	18.4 (4)
C8—C9—C10—C11	−0.6 (4)	C13—C18—C19—O3	−162.8 (2)
C7—C6—C11—C10	−0.3 (4)	C17—C18—C19—O4	−162.0 (2)
C7—C6—C11—C12	177.5 (2)	C13—C18—C19—O4	16.8 (3)
C9—C10—C11—C6	1.0 (4)		

### Hydrogen-bond geometry (Å, °)

**Table:**

*D*—H···*A*	*D*—H	H···*A*	*D*···*A*	*D*—H···*A*
N1—H1···O3^i^	0.86	2.01	2.824 (3)	157
N1—H1···O4^i^	0.86	2.44	3.171 (3)	144
O1—H1A···O4	0.82	1.77	2.582 (2)	169
N2—H2A···O4^i^	0.86	2.07	2.886 (3)	158
N2—H2B···O3^ii^	0.86	2.18	3.021 (3)	168
N3—H3A···O2	0.86	2.25	3.011 (3)	147
N3—H3B···O3^ii^	0.86	2.22	3.046 (3)	161
C3—H3···O2	0.93	2.36	3.141 (3)	142

Symmetry codes: (i) −*x*, *y*+1/2, −*z*+3/2; (ii) −*x*, −*y*+1, −*z*+2.
